# Treatment and Postinterventional Management of a Fusiform Intracranial Aneurysm in a Professional Soccer Player: A Case Report

**DOI:** 10.3389/fneur.2021.732640

**Published:** 2022-01-26

**Authors:** Manina Maja Etter, Leo Bonati, Ioannis Tsogkas, Gregor Hutter, Kristine Blackham, Raphael Guzman, Marios-Nikos Psychogios

**Affiliations:** ^1^Department of Neuroradiology, Clinic for Radiology and Nuclear Medicine, University Hospital Basel, Basel, Switzerland; ^2^Department of Neurology, University Hospital Basel, Basel, Switzerland; ^3^Department of Neurosurgery, University Hospital Basel, Basel, Switzerland

**Keywords:** intracranial aneurysms, professional athletes, endovascular treatment, dual antiplatelet therapy, case report

## Abstract

**Introduction:**

While intracranial aneurysms are common lesions affecting between 1 and 5% of the general population, the prevalence in professional athletes remains unknown. The result is uncertainty and lack of guidelines on appropriate treatment of these patients.

**Case Presentation:**

A 29-year-old professional soccer player presented in our hospital with an incidentally found intracranial aneurysm. After detailed depiction of the aneurysm and interdisciplinary discussion, endovascular treatment using a flow diverter was chosen to be the best treatment modality. Postinterventional medication consisted of dual antiplatelet therapy with aspirin and clopidogrel. The main challenge in managing the case of our patient was the combination of the dual antiplatelet treatment regime with his professional career in a contact sport.

**Conclusion:**

Due to lack of literature or similar reports regarding the management of professional athletes with intracranial aneurysms, the optimal treatment strategy remains unclear. Even though decisions should be made dynamically and case-adapted to each situation, developing a registry could help provide guidance and new ideas for similar cases in the future.

## Background

Intracranial aneurysms are common lesions affecting between 1 and 5% of the population, irrespective of ethnicity, or geographical location (1). There exists a considerable body of literature on the prevalence, diagnosis, and management of intracranial aneurysms in the general population, but there exist no studies that assessed the prevalence in professional athletes. The result is uncertainty and lack of guidelines on appropriate treatment, especially in terms of adjunctive antiplatelet treatment in cases of stent-assisted or flow diverter treatment and contact sports.

Formation of intracranial aneurysms is an incompletely understood, complex interplay between genetic and environmental risk factors. Hemodynamic stress and vascular risk factors, such as hypertension, lipid accumulation, arteriosclerosis, and smoking, are known to contribute to the formation of aneurysms in the second half of life. On the contrary, vascular risk factors do not seem to influence the development of aneurysms in younger patients. Therefore, conditions such as polycystic kidney disease, fibromuscular dysplasia, and connective tissue disorders have to be considered (1). The role of traumatic events, especially in athletes, should not be underestimated in the formation of intracranial aneurysms. A number of activities and mechanisms are known to potentially result in traumatic intracranial aneurysm (TICA), including blunt head trauma, which typically occurs during sports like soccer (2). Given the high intensity of the sport and acceleration forces to the brain and vessels while “heading the ball,” it seems evident that soccer carries an inherent risk of head injuries with possible consequences such as TICA. In the majority of cases, these injuries are the result of unexpected or unintentional contact with other players or the playing surface (3).

Here, we report the case of a professional soccer player who presented with an incidentally found intracranial aneurysm, and discuss our considerations regarding treatment, postinterventional medication, and his career.

## Case Presentation

**Figure 1:**
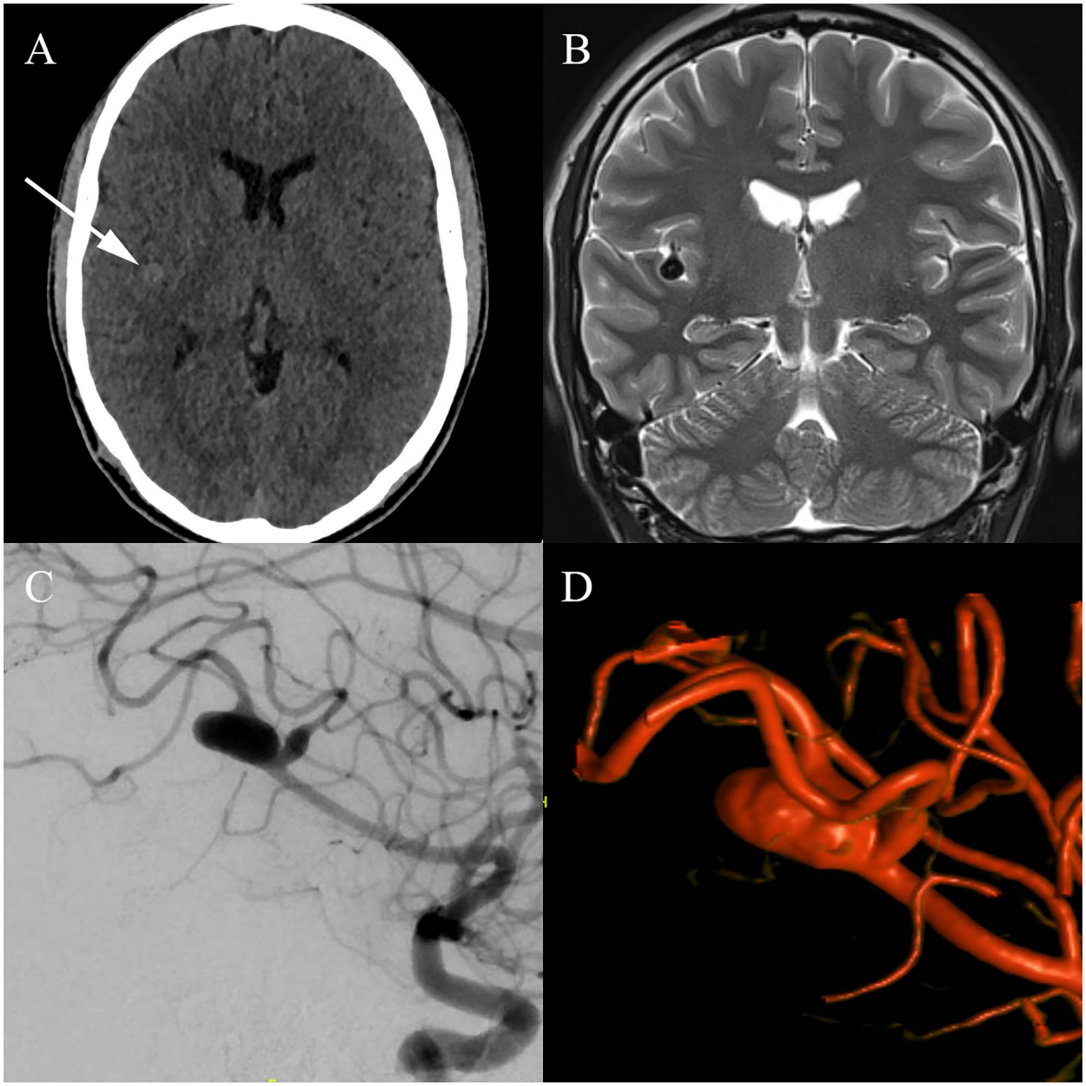
Initial imaging findings: **(A)** Initial computed tomography (CT) images reveal a hyperdense structure confined to the right Sylvian fissure, leading to further investigation with magnetic resonance imaging (MRI). **(B)** T2-weighted MRI images do not confirm the suspected hemorrhage; instead, a fusiform aneurysm located in the distal M2-segment of the right middle cerebral artery (MCA) is delineated. **(C)** Cerebral angiogram illustrates a fusiform aneurysm, located in the inferior trunk of the right MCA. Furthermore, fusiform dilatation of a parieto-occipital branch arising from the right MCA is seen. The lesions are located adjacent to the right insula, in the depth of the Sylvian fissure. **(D)** Three-dimensional volume-rendering technique (VRT) of the aneurysm.

**Figure 2:**
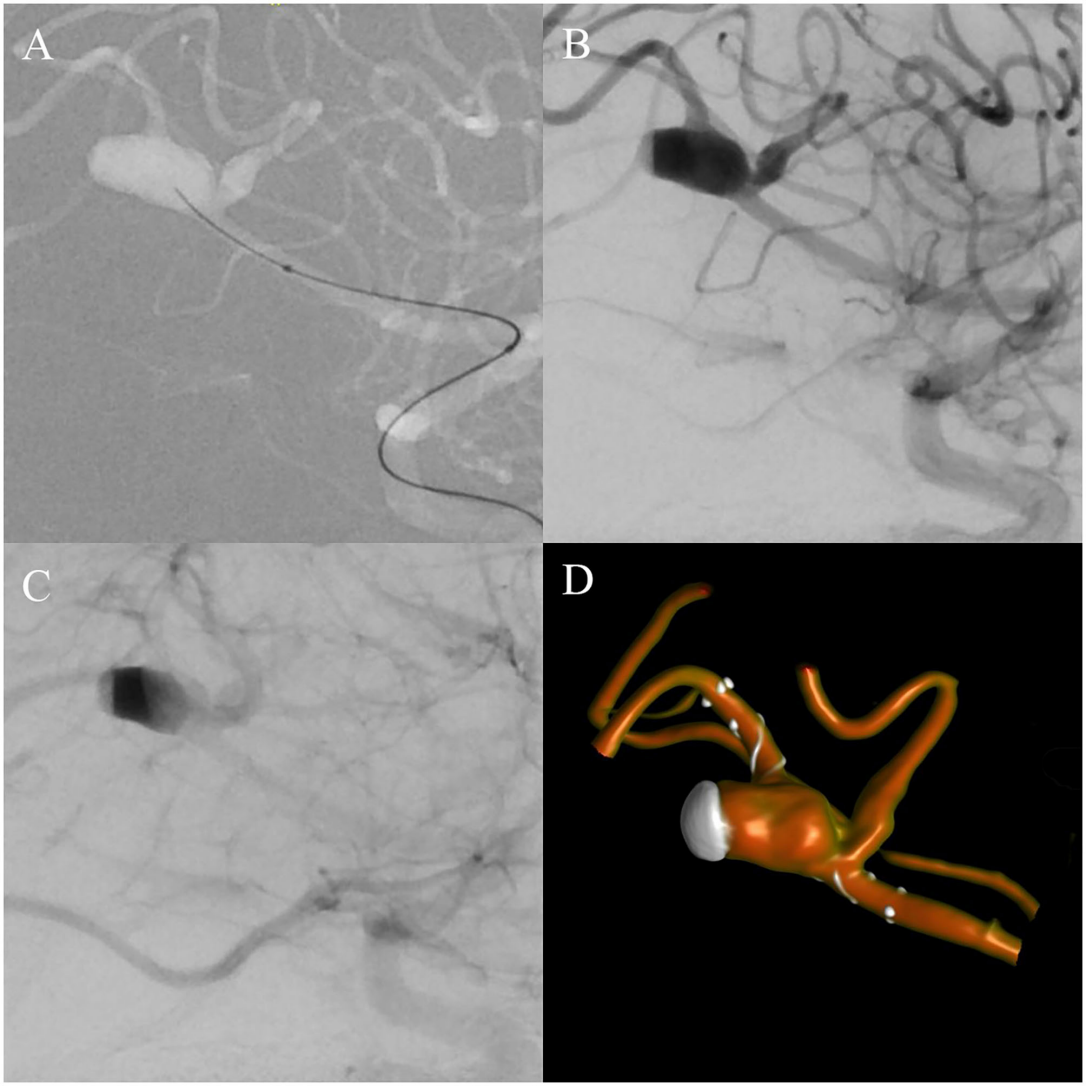
Intra-interventional cerebral angiograms: **(A)** Lateral working projection during navigation of the microcatheter and microwire toward the aneurysm. **(B)** Directly after implantation of the flow diverter, contrast media stasis can be seen at the dome of the aneurysm as a gray, subtracted cap in the arterial phase. **(C)** In the venous phase, additional stasis in the majority of the aneurysm sack is depicted as a black band. **(D)** Postinterventional three-dimensional VRT showing the positioning of the flow diverter in relation to the aneurysm and marked stasis of contrast medium at the dome of the aneurysm.

**Figure 3:**
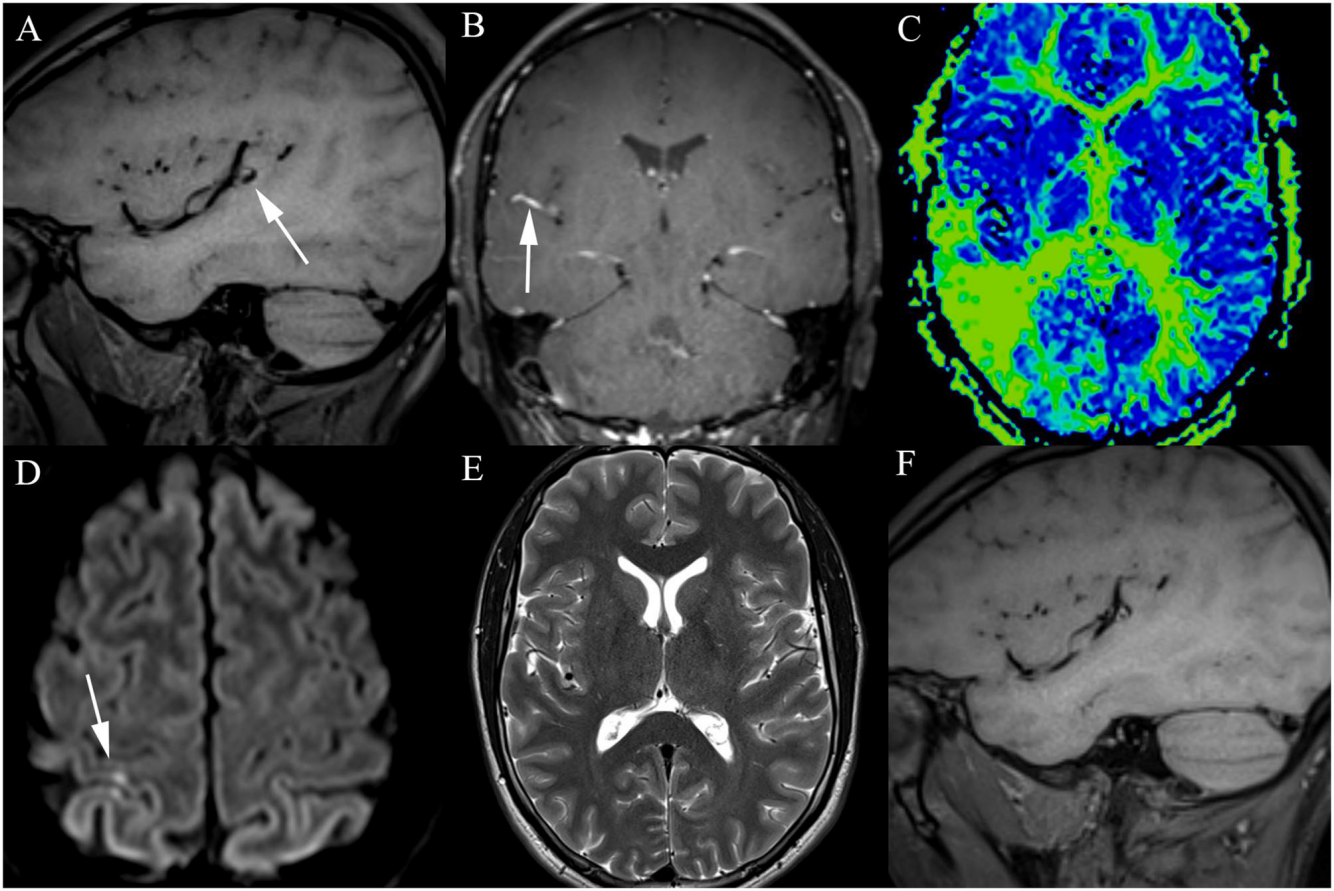
Postinterventional imaging findings: **(A)** T1-weighted image 6 months after flow diverter implantation showing hyperintense thrombotic material around the flow diverter and near-complete occlusion of the aneurysm. The initial severe stasis at the dome (Figure 2B) is depicted as a hypointense cap. **(B)** Slow flow in the side branch originating from the aneurysm wall is documented 6 months post intervention after changing to monotherapy with aspirin. **(C)** Perfusion deficit of the affected territory is visible on mean transit time reconstructions of the MRI perfusion. **(D)** Small ischemic lesions, located in the right superior parietal lobule, are seen on DWI. **(E)** One month after detection of the slow flow in the side branch of the aneurysm-wall, T2-weighted images did not reveal an infarction in areas with delayed perfusion. **(F)** As 12 month-follow-up MRIs reveal, the aneurysm is completely filled with a hyperintense thrombotic material, which means that full occlusion of the aneurysm was achieved.

In August 2019, while trying to head a ball, a 29-year-old professional soccer player sustained a concussion while colliding with another player. On arrival in the emergency room, the patient presented with retrograde amnesia and a Glasgow Coma Scale (GCS) 15 without any focal neurological deficits. Neuroradiologists suspected a traumatic subarachnoidal hemorrhage on computed tomography (CT) scans, confined to the Sylvian fissure (Figure 1A). Further imaging with magnetic resonance imaging (MRI) was recommended and showed a 10 × 6 × 5 mm fusiform aneurysm of the right middle cerebral artery (MCA), located in the distal M2-segment (Figure 1B). For exact depiction of the incidental aneurysm and assessment of treatment options, cerebral angiograms were acquired (Figures 1C,D). After interdisciplinary discussion, endovascular treatment using a FRED Jr. flow diverter (2.5 × 20 × 25 mm) was chosen to be the best treatment modality. The procedure was performed by the end of August 2019 (Figure 2), and the patient recovered uneventfully. Postinterventional medication consisted of dual antiplatelet therapy with aspirin 100 mg per day and clopidogrel 75 mg per day. In February 2020, in order to be cleared for contact drills and regular games, we stopped clopidogrel therapy. A few days after changing to monotherapy with aspirin, he presented to the emergency department with progressive headache, nausea, and vomiting. Clinical examination revealed no focal neurological deficits and a GCS of 15. Brain MRI indicated nearly complete thrombosis of the aneurysm (Figure 3A). Slow-flow was depicted in an MCA side branch (Figure 3B), originating from the aneurysm-wall, with a perfusion deficit of the respective territory (Figure 3C) and small embolic lesions on diffusion-weighted imaging (DWI, Figure 3D). Therefore, we decided to restart the dual antiplatelet therapy until the next follow-up 1 month later, and limit sport activities to non-contact drills. The follow-up MRI scan showed no new cerebral infarction (Figure 3E) and normalization of the flow in the MCA side branch. After consulting with our neurologists, we continued with clopidogrel monotherapy (75 mg per day), stopped aspirin, and cleared the patient for professional games. Six-month follow-up MRI scan showed complete occlusion of the aneurysm (Figure 3F), regular flow in the side branch, and no cerebral infarction in the MCA territory or any hemorrhages. The patient is currently participating in soccer practice and games without any problems.

## Discussion and Conclusion

After the detection of an unruptured intracranial aneurysm, various factors have to be considered to identify the ideal management. In general, there are three options to treat intracranial aneurysms: craniotomy with clip ligation, endovascular approaches, and observation. Incidentally discovered aneurysms are usually observed or treated electively, depending on the size and location of the aneurysm as well as patient-related factors, such as age, health, and professional conditions (4). Selecting the best treatment modality is even more difficult in professional athletes, among other things, because of lack of medical literature for guidance on appropriate postinterventional medication in cases of flow diverter implantation.

In our case, the best option was considered to be treatment with a mini flow diverter, which features the necessity of adjunctive antiplatelet medication. The same medication regimen applies to stent-assisted endovascular treatment, which represents a disadvantage of these two strategies because of the distal location and fusiform nature of the aneurysm. Alternatives of clip ligation and coiling were interdisciplinary discussed because of their advantage of not necessitating antiplatelet medication but were not feasible in our patient's case because of the fusiform nature of the aneurysm and its localization within the Sylvian fissure.

The main challenge in managing the case of our patient was the combination of aspirin and clopidogrel with his professional career in a contact sport. With flow diverter-treated intracranial aneurysms, it is impossible to omit antiplatelet drugs periinterventionally. Lacking established guidelines, individualized, case-adapted decisions had to be taken in the following months. We aimed at enabling monotherapy as soon as possible while simultaneously ensuring high-enough antithrombotic drug activity to clear him for games and contact drills. As we used a small flow diverter in a distal vessel, we deemed it reasonable to sustain the dual antiplatelet therapy for 6 months and then continue with the aspirin monotherapy, independent of the profession of our patient. However, after experiencing a perfusion deficit and slow flow in the aneurysm side branch, the dual antiplatelet therapy was reinstated for 1 more month. After 4 weeks, and in order to clear the patient for games, we tried a different monotherapy regimen, this time with clopidogrel. The hypothesis was that clopidogrel alone would feature higher potency in terms of preventing arterial thrombosis compared to aspirin monotherapy. This plan was successful, as the patient could participate in games without any hemorrhagic complications, and we did not observe any infarctions or slow flow in the side branch in follow-up imaging. Our main concern was to not let our patient participate in soccer practice and games as long as he is under the dual antiplatelet therapy.

Regarding the formation of the aneurysm in our patient, it is unlikely that vascular risk factors, such as hypertension, lipid accumulation, and arteriosclerosis, contributed to the process. Conditions known to be associated with intracranial aneurysms in young patients, such as fibromuscular dysplasia, were evaluated and ruled out. Trauma-associated head and brain injuries, especially repetitive trauma, can result in hemorrhages, edema, alterations in cerebral blood flow, vasospasm, dissection, blood brain barrier disruption, chronic inflammation, and vessel wall degeneration, which in turn can facilitate the formation of aneurysms (5, 6). The traumatic event that caused the presenting concussion in our patient was not related to the aneurysm, as we did not observe any intramural hematoma on initial MRI. Regarding the shape of the aneurysm, a mycotic aneurysm could be considered, as these aneurysms are defined by wall dilatation due to infection. However, the risk is higher in immune-compromised hosts; therefore, the pathophysiology of mycotic aneurysms does not match the history of our patient (7). The actual origin of the aneurysm of our patient remains unclear, but the most likely etiology is due to repetitive blunt head injuries during soccer practice and games, leading to a dissecting aneurysm that grew over time and took the final form we observed in August 2019.

Beyond medical considerations, public relations and financial aspects may affect the final decision in the treatment of aneurysms in professional athletes. While we, as physicians, base our decisions on medical evidence and literature, other aspects such as financial circumstances, the situation, or the wish of the athlete or the soccer club may influence the final decision of the patient. The case of our patient was complicated by the fact that his aneurysm was incidentally detected during his contract year. Therefore, while choosing a prompt treatment of the aneurysm, he also asked for the option to be cleared for games and contact drills as soon as possible.

During the last few years, newer surface-coated flow diverters, which limit the thrombotic potential of stents, have emerged. These surface-modified devices are ideally used for ruptured dissecting aneurysms but could also be useful in cases like ours. They may not require dual antiplatelet therapy and could be combined with monotherapy and continuous testing for antithrombotic drug activity. Hence, this approach could allow professional athletes to start with contact drills and games soon after the interventional procedure (8, 9).

We present the rare case of a professional soccer player, diagnosed with a fusiform MCA aneurysm, necessitating treatment with a flow diverter and subsequent dual antiplatelet medication. The history of our patient is, therefore, noteworthy in view of lacking literature or similar reports regarding the management of professional athletes under these circumstances. The situation becomes even more complex when the athlete wishes to continue his career despite the need for antiplatelet medication after endovascular aneurysm treatment. Developing a registry of professional athletes requiring antiplatelet medication, or even anticoagulation, could help to provide guidance and promote new ideas for similar cases in the future. Since the management of this case was successful, we wanted to provide advice and ideas on case-adapted decision-making for similar cases in the future. On a final note, we can say that decisions in these cases should be made dynamically and case-adapted to the situation, as there are more than the medical aspects at play in this arena.

## Data Availability Statement

The original contributions presented in the study are included in the article/Supplementary Material, further inquiries can be directed to the corresponding author.

## Ethics Statement

Written informed consent was obtained from the individual(s) for the publication of any potentially identifiable images or data included in this article.

## Author Contributions

ME: data acquisition and drafting the manuscript. LB, IT, GH, KB, and RG: revision of the manuscript. M-NP: data interpretation, supervision, and critical revision of the manuscript. All authors read and approved the final version of the manuscript.

## Conflict of Interest

The authors declare that the research was conducted in the absence of any commercial or financial relationships that could be construed as a potential conflict of interest.

## Publisher's Note

All claims expressed in this article are solely those of the authors and do not necessarily represent those of their affiliated organizations, or those of the publisher, the editors and the reviewers. Any product that may be evaluated in this article, or claim that may be made by its manufacturer, is not guaranteed or endorsed by the publisher.

## Abbreviations

TICA: traumatic intracranial aneurysm
GCS: Glasgow Coma Scale
CT: computed tomography
MRI: magnetic resonance imaging
MCA: middle cerebral artery
VRT: volume-rendering technique
DWI: diffusion-weighted imaging.
