# Supplementary material for: Treatment and Postinterventional Management of a Fusiform Intracranial Aneurysm in a Professional Soccer Player: A Case Report
Source: Front Neurol. 2022 Jan 26;12:732640. doi: 10.3389/fneur.2021.732640 (PMC8825373; doi:10.3389/fneur.2021.732640)
Supplement: Supplementary file 1 [file Data_Sheet_1.PDF]

CARE case report guidelines

CARE Checklist of information to include when writing a case report

(cc) BY-NC-ND

✓

Topic	Item	Checklist item description	Reported on Line
Title	1	The diagnosis or intervention of primary focus followed by the words "case report"	1 - 2
Key Words	2	2 to 5 key words that identify diagnoses or interventions in this case report, including "case report"	20 - 21
Abstract (no references)	3a	Introduction: What is unique about this case and what does it add to the scientific literature?	24 - 26
	3b	Main symptoms and/or important clinical findings	27 - 28
	3c	The main diagnoses, therapeutic interventions, and outcomes	28 - 32
	3d	Conclusion—What is the main "take-away" lesson(s) from this case?	33 - 36
Introduction	4	One or two paragraphs summarizing why this case is unique (**may include references**)	39 - 45
Patient Information	5a	De-identified patient specific information.	64
	5b	Primary concerns and symptoms of the patient.	64-67, 78-79
	5c	Medical, family, and psycho-social history including relevant genetic information	64-90
	5d	Relevant past interventions with outcomes	-
Clinical Findings	6	Describe significant physical examination (PE) and important clinical findings.	65-67, 78-79
Timeline	7	Historical and current information from this episode of care organized as a timeline	-
Diagnostic Assessment	8a	Diagnostic testing (such as PE, laboratory testing, imaging, surveys).	68-90
	8b	Diagnostic challenges (such as access to testing, financial, or cultural)	-
	8c	Diagnosis (including other diagnoses considered)	69-70, 80-82
	8d	Prognosis (such as staging in oncology) where applicable	-
Therapeutic Intervention	9a	Types of therapeutic intervention (such as pharmacologic, surgical, preventive, self-care)	72-90
	9b	Administration of therapeutic intervention (such as dosage, strength, duration)	72-90
	9c	Changes in therapeutic intervention (with rationale)	83
Follow-up and Outcomes	10a	Clinician and patient-assessed outcomes (if available)	87-90
	10b	Important follow-up diagnostic and other test results	87-90
	10c	Intervention adherence and tolerability (How was this assessed?)	73-90
	10d	Adverse and unanticipated events	79-84
Discussion	11a	A scientific discussion of the strengths AND limitations associated with this case report	92-147
	11b	Discussion of the relevant medical literature **with references**.	92-147
	11c	The scientific rationale for any conclusions (including assessment of possible causes)	148-157
	11d	The primary "take-away" lessons of this case report (without references) in a one paragraph conclusion	148-157
Patient Perspective	12	The patient should share their perspective in one to two paragraphs on the treatment(s) they received	136-142
Informed Consent	13	Did the patient give informed consent? Please provide if requested	Yes ☒ No ☐
